# [Corrigendum] PF‑2341066 combined with celecoxib promotes apoptosis and inhibits proliferation in human cholangiocarcinoma QBC939 cells

**DOI:** 10.3892/etm.2024.12399

**Published:** 2024-01-24

**Authors:** Chen Chen, Qinghua Zeng, Liang Luo, Chengzhi Cai

Exp Ther Med 15:4543–4549, 2018; DOI: 10.3892/etm.2018.5967

Subsequently to the publication of the above article, the authors drew to the Editor’s attention that the western blots shown in Fig. 2H and I had been represented using the same control western blots, whereas these figure parts should have been shown incorporated in the same figure panel (now Fig. 2H; see the revised figure opposite). Moreover, the second-listed author (Dinghua Yang) requested that their name be removed from the article, with the agreement of all the other authors. Consequently, the corresponding author on this paper is now the first-named author, Chen Chen. The revised author affiliation details are therefore as follows:

CHEN CHEN^1^, QINGHUA ZENG^2^, LIANG LUO^1^ and CHENGZHI CAI^1^

^1^Department of Hepatobiliary Surgery; ^2^Department of Rheumatology and Immunology, Hunan Provincial People’s Hospital, Changsha, Hunan 410002, P.R. China

*Correspondence to*: Dr Chen Chen, Department of Hepatobiliary Surgery, Hunan Provincial People’s Hospital, 61 Jiefang West Road, Changsha, Hunan 410002, P.R. China E-mail: chenchen2043@163.com

Note that the error associated with the assembly of Fig. 2 did not have a major impact on either the overall results or on the conclusions reported in this study. All the authors agree with the publication of this corrigendum, and are grateful to the Editor of *Experimental and Therapeutic Medicine* for granting them the opportunity to publish this; furthermore, they apologize to the readership for any inconvenience caused.

## Figures and Tables

**Figure 2:**
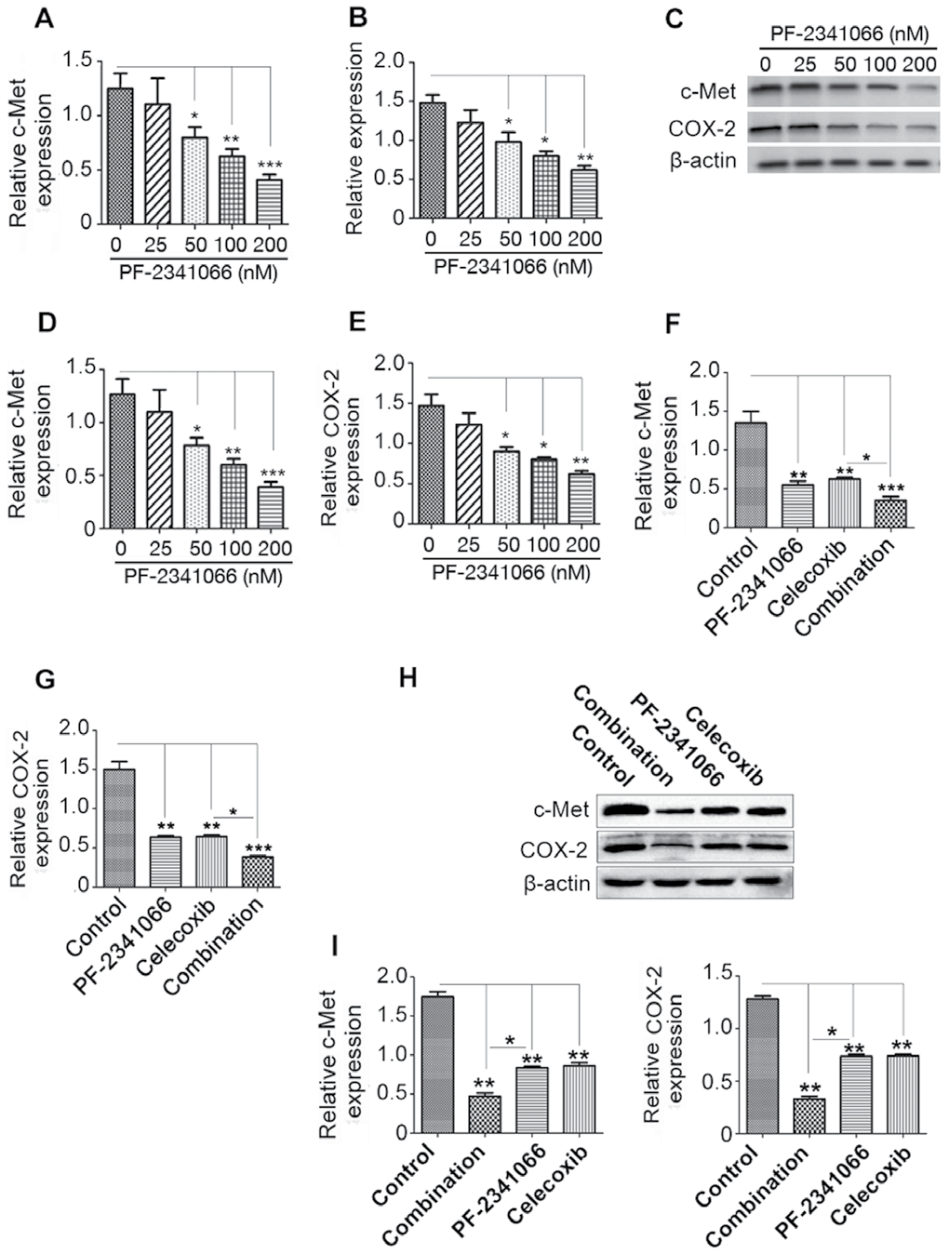
Expression of c-Met and COX-2 in human cholangiocarcinoma QBC939 cells following PF-2341066 and celecoxib treatment. The expression of (A) c-Met and (B) COX-2 mRNA following treatment with 0, 25, 100 and 200 nM PF-2341066 was measured using RT-qPCR. (C) Western blotting was performed to measure the protein expression of (D) c-Met and (E) COX-2 following treatments. (F) c-Met and (G) COX-2 mRNA expression following treatment with PF-2341066 and celecoxib was measures using RT-qPCR. (H and I) Western blotting was performed to measure the expression of c-Met and COX-2 following treatment. ^*^P<0.05, ^**^P<0.01 and ^***^P<0.001. COX-2, cyclooxygenase-2; RT-qPCR, reverse transcription-quantitative polymerase chain reaction; Combination, treatment with 100 nM PF-2341066 + 100 µM celecoxib.

